# DEVELOPMENTAL EXPOSURE TO THE PARKINSON’S DISEASE-ASSOCIATED ORGANOCHLORINE PESTICIDE DIELDRIN ALTERS DOPAMINE NEUROTRANSMISSION IN A-SYNUCLEIN PRE-FORMED FIBRIL (PFF)-INJECTED MICE

**DOI:** 10.51894/001c.123058

**Published:** 2024-08-30

**Authors:** Sierra L. Boyd

**Affiliations:** 1 College of Osteopathic Medicine Michigan State University; 2 Department of Translational Neuroscience, Grand Rapids Michigan State University

## 70

### INTRODUCTION/BACKGROUND

Parkinson’s disease (PD) has emerged as the fastest-growing neurological disorder globally, exhibiting a rise that surpasses the effects of aging and intensifies particularly in recently industrialized regions, pointing towards potential environmental influences. Accumulating evidence from epidemiological, post-mortem, and mechanistic studies underscores the association between persistent organic pollutants, notably the organochlorine pesticide dieldrin, and an elevated risk of PD.

### OBJECTIVES/HYPOTHESIS

In murine models, developmental exposure to dieldrin induces a male-specific augmentation in neuronal susceptibility to 1-methyl-4-phenyl-1,2,3,6-tetrahydropyridine (MPTP) and synucleinopathy. Specifically, utilizing the α-synuclein pre-formed fibril (PFF) model, this exposure results in heightened deficits in striatal dopamine (DA) turnover and motor impairments on challenging beams. Our hypothesis posits that changes in DA handling contribute to these observed effects, prompting an investigation into vesicular monoamine transporter 2 (VMAT2) function and DA release in this dieldrin/PFF dual-hit model.

### METHODS

Female C57BL/6 mice underwent exposure to 0.3mg/kg dieldrin or vehicle every 3 days through feeding, commencing at 8  weeks of age and persisting throughout breeding, gestation, and lactation. Male offspring, derived from distinct litters, received unilateral intrastriatal injections of α-syn PFFs at 12 weeks of age. Subsequently, vesicular 3H-DA uptake assays and fast-scan cyclic voltammetry were conducted 4 months post-PFF injection.

### RESULTS

Dieldrin-induced an elevation in DA release in striatal slices in PFF-injected animals, without concomitant changes in VMAT2 activity. These results indicate that developmental dieldrin exposure elicits a compensatory response to synucleinopathy-triggered striatal DA loss.

### CONCLUSION

Our findings align with the concept of silent neurotoxicity, wherein developmental exposure to dieldrin primes the nigrostriatal striatal system, leading to an exacerbated response to synucleinopathy. This occurs in the absence of observable alterations in typical markers of nigrostriatal dysfunction and degeneration, shedding light on potential mechanisms underlying environmental contributions to PD pathogenesis.

